# In Silico Selection and In Vitro Evaluation of New Molecules That Inhibit the Adhesion of *Streptococcus mutans* through Antigen I/II

**DOI:** 10.3390/ijms22010377

**Published:** 2020-12-31

**Authors:** Raúl E. Rivera-Quiroga, Néstor Cardona, Leonardo Padilla, Wbeimar Rivera, Cristian Rocha-Roa, Mayri A. Diaz De Rienzo, Sandra M. Morales, María C. Martinez

**Affiliations:** 1 Group of Investigation in Oral Health, Faculty of Dentistry, Antonio Nariño University, Av. Bolívar # 49 North-30, Armenia 630001, Quindío, Colombia; nestorcardonape@uan.edu.co; 2 GYMOL Group, Faculty of Health Sciences, Quindío University, Street 12N, Armenia 630001, Quindío, Colombia; lpadilla@uniquindio.edu.co; 3 Oral Microbiology Laboratory, Faculty of Dentistry, University of Antioquia, 64 Street No. 52–59, Block 31, No. 216, Health Area, Medellin 050001, Antioquia, Colombia; wbeimar.rivera@udea.edu.co (W.R.); sandra.morales@udea.edu.co (S.M.M.); mcecilia.martinez@udea.edu.co (M.C.M.); 4 GEPAMOL Group Faculty of Health Sciences, Quindío University, Street 12N, Armenia 630001, Quindío, Colombia; ccrochar@uqvirtual.edu.co; 5 School of Pharmacy and Biomolecular Sciences, Liverpool John Moores University, James Parsons Building 10.05C, Byrom Street, Liverpool L3 3AF, UK; m.a.diaz@ljmu.ac.uk

**Keywords:** *Streptococcus mutans*, adhesion proteins, antigen I/II, dental caries, structure-based virtual screening, molecular dynamics

## Abstract

*Streptococcus mutans* is the main early colonizing cariogenic bacteria because it recognizes salivary pellicle receptors. The Antigen I/II (Ag I/II) of *S. mutans* is among the most important adhesins in this process, and is involved in the adhesion to the tooth surface and the bacterial co-aggregation in the early stage of biofilm formation. However, this protein has not been used as a target in a virtual strategy search for inhibitors. Based on the predicted binding affinities, drug-like properties and toxicity, molecules were selected and evaluated for their ability to reduce *S. mutans* adhesion. A virtual screening of 883,551 molecules was conducted; cytotoxicity analysis on fibroblast cells, *S. mutans* adhesion studies, scanning electron microscopy analysis for bacterial integrity and molecular dynamics simulation were also performed. We found three molecules ZINC19835187 (ZI-187), ZINC19924939 (ZI-939) and ZINC19924906 (ZI-906) without cytotoxic activity, which inhibited about 90% the adhesion of *S. mutans* to polystyrene microplates. Molecular dynamic simulation by 300 nanoseconds showed stability of the interaction between ZI-187 and Ag I/II (PDB: 3IPK). This work provides new molecules that targets Ag I/II and have the capacity to inhibit in vitro the *S. mutans* adhesion on polystyrene microplates.

## 1. Introduction

In 2016, dental caries was classified as the most prevalent pathology in the world, affecting 2.4 billion people [1,2]. This pathology is among the oral diseases related to oral microbiota alteration [3], characterized by perforations or structural damage of the teeth, called carious lesions [4]. There are three well-known risk factors for the development of caries: personal factors that are related to socioeconomic status—i.e., dental insurance coverage, attitudes and knowledge about oral health and oral hygiene; oral environmental factors such as saliva, fluoride, chewing gum, pH, bacteria, calcium, phosphates, proteins and factors that directly contribute to the development of caries, such as, the tooth, diet (consumption of sugars), bacterial biofilms and time [5].

Oral microorganisms that cannot adhere to a surface are transported by salivary flow out of the mouth and into the digestive tract, but many oral bacteria possess mechanisms of adherence to solid surfaces (co-adhesion), such as coated teeth from salivary films, to squamous surfaces such as epithelial tissue or bacteria that are attached to the surface (co-aggregation) [6]. The streptococci compete for adhesion binding sites on the saliva-coated tooth surface and are able to produce antimicrobial compounds and *S. mutans* can become dominant in oral biofilms, leading to dental caries development [7]. This organism also produces glycosyltransferases (gtfs), multiple glucan-binding proteins (Gbps), antigen I/II (also called SpaP, Pac, P1), and collagen-binding protein, these surface proteins coordinate the production of dental plaque [8]. The ability to form biofilms is one of the main *S. mutans* characteristics and it is a complex process of protein–bacterium interaction that begins with the attachment of a single cell, aggregation, microcolony formation until a mature biofilm [9]. Adherence to host tissues represents a critical step in the pathogenic process and is usually mediated by bacterial surface-exposed proteins in which *S. mutans* have mechanisms for adhesion sucrose-dependent (Gtfs essential) and sucrose-independent (Ag I/II essential) [10].

In the absence of sucrose, *S. mutans* synthesizes several important adhesins such as antigen I/II (also called SpaP, Pac), which specifically binds to a glycoprotein called salivary agglutinin (SAG) [11,12], which has been proposed that participates as well in the tooth bacterial adhesion [13], and biofilm formation; this due to the fact that Ag I/II-deficient mutants formed 65% less biofilm than the wild-types [14] and a decrease in its ability to promote the aggregation and invasion of the dentin of the collagen-dependent tooth [8,14]. Ag I/II virulence has been evaluated in a gnotobiotic rat model [15] and has been considered a promising target antigen for anticaries vaccines [16,17,18,19]. The overall structure of antigen I/II is conserved in all members of this protein family; this multidomain protein is composed of 1500–1566 amino acid residues (140- to 180-kDa) with a structure composed of alanine-rich variable V, proline-rich P, and C-terminal C domains [19,20,21]. The antigen I/II family of adhesins is cell wall-associated polypeptides that are widely distributed on the cell surface of many streptococci and is not only important for initial streptococcal adhesion to the host but also for inter-bacterial adhesion and “secondary” colonization; it also mediates interactions between *S. mutans* and *Candida albicans* [22,23]. Additionally, the presence of these proteins on the cell surface determines the adherence of *S. mutans* to SAG, but no difference in SAG-mediated adherence could be seen between type A and B strains [24]. Analysis of host and bacterial phenotype variation in adhesion of *S. mutans* has determined that the host saliva phenotype and Ag I/II V-region plays a prominent role [25]. However, crystal structure information shows a possible model for AgI/II binding to SAG, where interactions occur at both the distal end through the A3VP1 region, and at a secondary adherence site mediated by the C-terminal domain [20].

Numerous therapies for dental caries have been proposed apart from the extensive use of fluoride, as xylitol [26,27], chlorhexidine [28,29], immunization [16,30], molecules derived from natural products [31], metal ions or oxidizing agents and even antibodies that specifically bind to *S. mutans* targets (GtfB, GtfC, GtfD, Ag I/II) inhibiting the bacterial ability to develop biofilms [18,32], strategies have been reported to target specific caries pathogens or to indiscriminately eliminate oral microbiota. However, the effectiveness of these methods is yet to be recognized, and safety concerns have been raised with regard to their negative impact on the ecology of the oral microbiota [33]. Other potential anticariogenic methods include the casein phosphopeptide-amorphous calcium phosphate nanocomplex (CPP-ACP), an agent that saturates saliva and biofilm, favoring the dental remineralization [34]; arginine which inhibits the growth of acidogenic or aciduric bacteria by raising the pH of the oral environment sugar substitutes [33]; it has been proposed that the combined antimicrobial effect of arginine and fluoride has a potential synergistic effect in maintaining an eco-friendly oral microbial equilibrium which helps prevent tooth decay, though the mechanism of arginine over the destabilization of biofilm is not yet clear [35,36]. These promising approaches may include the use of arginine as prebiotic and selected bacterial strains with an arginine deiminase pathway (ADS+) as a probiotic, such as *Streptococcus dentisani*, a bacterial isolated from dental plaque of caries-free individuals that has been shown to have several beneficial effects in vitro which could contribute to promote oral health, including an antimicrobial activity against oral pathogens by the production of bacteriocins and a pH buffering capacity through ammonia (produced by arginine deiminase system), and the topic application of these probiotic could decrease the amount of dental plaque, but no differences were observed in the placebo group [37].

Few of these treatments have been proven to confer selectivity against *S. mutans* or other cariogenic bacteria to prevent caries without disturbing the ecological balance between pathogens and commensal bacteria in the oral cavity. In recent decades, the virtual search for inhibitors based on structures has taken an interest in drug discovery [38,39,40]; for oral microbiology, the use of this strategy is relatively new, particularly in the cariogenic context, but several proteins have been proposed that could be used as inhibitory molecules [41]. Gtf-C has been used as a target on the search of molecules with affinity to this protein and for selectively inhibition of *S. mutans* biofilms formation mainly due to the ability to inhibit the synthesis of exopolysaccharides (EPS) in vitro, the biofilm formation and reduce in vivo the caries incidence and severity in a rat model [42,43]. Although the Ag I/II adhesin has been reported to play an important role in the early stages of *S. mutans* biofilm development, participating in adhesion and co-aggregation with other bacteria and fungi such as *C. albicans*, there are no reports of computational studies that use this protein as a target; therefore, the aim of this work is to identify in silico molecules with an inhibitory effect on *S. mutans* Ag I/II, which have no cytotoxic activity on human cells.

## 2. Results and Discussion

### 2.1. Structure-Based Virtual Screening

#### 2.1.1. Target Proteins Selection

**Figure 1:**
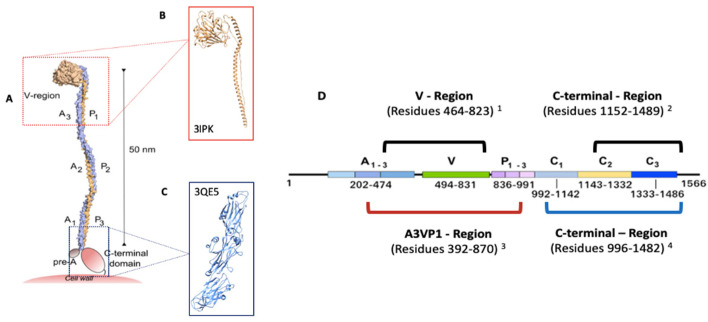
Crystals structures from the *S. mutans* Ag I/II protein (**A**). Model of *S. mutans* Ag I/II structure and predicted binding with human SAG (taken from [21]). (**B**) Crystal structure of A3VP1 protein from Ag I/II (3IPK) [21]. (**C**). Crystal structure C-domain protein from the Ag I/II (3QE5) [20]. (**D**). Schematic representation of the Ag I/II protein sequence (1566 aa) (Taken from [20]) and the description of the aa residues that constitute the crystals structures of the protein regions: 1—region V (PDB: 1JMM) [44]; 2—C-terminal domain [45]; 3—A3VP1 region (PDB: 3IPK) [21] and 4—the C-terminal region (PDB: 3QE5) [20].

The Ag I/II of *S. mutans* play an essential role in the etiology and pathogenesis of dental caries. Therefore, the discovery of inhibitors of Ag I/II may facilitate the development of drugs that prevent dental caries. Ag I/II is among the cell wall-anchored adhesins that mediates attachment of *S. mutans* to tooth surfaces, recognize salivary glycoproteins, and are also involved in biofilm formation. Finding small molecules that bind to the Ag I/II may interfere with the function of these adhesins. To address this, we selected a sequence of the *S. mutans* Ag I/II adhesin, and we found a crystal structure from different regions of AgI/II, which corresponds to the A3VP1 region (PDB: 3IPK) [21], C-terminal domain, region V (PDB: 1JMM) [44] and two from the C-terminal domain (PDB: 3QE5) [20] and (PDB: 3OPU) [45] (Figure S1). According to sequence similarity and coverage results (Table S1), the crystals structures of the proteins 3IPK and 3QE5 were selected, due to their role in *S. mutans* adhesion to the tooth, through their SAG binding [21] (Figure 1).

Sequence and structural similarity analysis of 3IPK and 3QE5 was performed in order to identify other similar proteins in another organism. However, no significant homologies were found, which could be an indication that these molecules would not affect human (Table S2) or bacterial proteins important for the ecological balance of the oral microbiota, but a lack of overall protein similarity may not exclude local similarities in the properties of the ligand binding pockets—i.e., two unrelated proteins may share pockets with the ability to bind a common compound. On the other hand, we found sequence homologies with proteins from 23 bacterial species, which are mostly normal inhabitants of the oral cavity, gastrointestinal and genitourinary tracts, but are associated with different pathologies (Tables S3 and S4). Regarding the structural homologies for 3IPK and 3QE5 proteins, there were no similarities with any human or bacterial proteins, only with *S. mutans* Ag I/II regions, A3VP1 region (PDB: 3ioxA) [21] and N-terminal and C-terminal interaction complex (PDB: 4tshA) (Table S5) [46].

A fundamental step for the search of molecules based on virtual structures, is the pocket selection, these sites must have typical characteristics such as concave, have a variety of hydrogen bridge donors and acceptors and hydrophobic characteristics [47]; otherwise, false negatives may occur when selecting molecules for in vitro assays, since errors may occur when there are no binding sites in the protein or when homology models are used, causing for example, small-volume pockets to be selected that will generate incorrect unions or conformations [48]. For that reason, in this study, two multiservers or specific programs were used for protein ligand binding site prediction, which have different selection algorithms; the MetaPocket uses a consensus method based on the predicted sites of four free access programs LIGSITEcs, PASS, Q-SiteFinder and SURFNET, which are combined to improve the success rate of the prediction and which is based on the geometry and surface of the proteins [49], unlike the COACH that uses the consensus of two methods, one based on the comparison of specific binding substructures (TM-SITE) and the other on the alignment of the sequence profile (S-SITE), for predictions of binding sites based on known proteins [50] (Figure S2).

#### 2.1.2. Molecule Selection

Ten molecules were arbitrarily selected according to their molecular docking score and ten according to the number of pockets interaction. The lowest docking score indicates high affinity between the molecule and the ligand. Therefore, molecules that interacted in several binding sites were selected, because those could have a greater coating of the target protein allowing the inhibition of the two important regions of Ag I/II involved in its adhesin function. Interestingly, we found molecules that bind to more than one site of the same protein domain, A3VP1 in the V region and C-terminal region; this could have been possible because these proteins have several conserved regions in their structure; in PDB: 3IPK the A region typically consists of 3–4 alanine rich repeats (82 residues each) with 23–30% alanine content; the P region has 3–4 proline-rich repeats (39 residues each) with ~35% proline content and in PDB: 3QE5 the sequence have a high proline content that forms a repetitive proline-rich region [19,20,21]. In addition, A3VP1 and the C-terminal fragments have multiple binding sites and similar affinities for binding to SAG, which support the simple proposed model that the high-affinity binding of AgI/II with SAG occurs via the apical fishhook-like structure observed within A3VP1, and an additional interaction occurring within the C-terminal region [21].

Finally, nineteen molecules were obtained, because the ZINC19924906 molecule from the library “Small” was selected according to the best docking score and the interaction with different ligand sites in the domains. The molecules with the lowest docking score were from the library “Natural”, such as ZINC68568370 with a docking score of −12.8. The molecules that had a higher number of binding sites were found from the library “Small” and were coupled to the 12 pockets used for docking, which means that they have affinity for several pockets in both the PDB domain: 3IPK and PDB: 3QE5 (Table 1).

**Figure 2:**
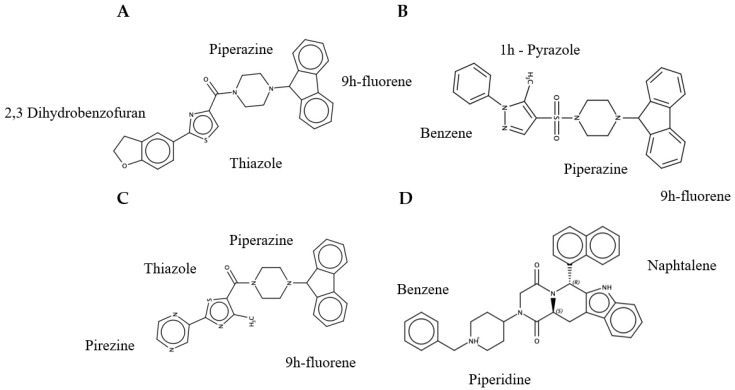
Molecules structures selected by in silico analysis, which have affinity for Ag I/II and inhibitory potential of *S. mutans* adhesion. (**A**)-ZI-187; (**B**)-ZI-939; (**C**)-ZI-906; (**D**)-ZI-498.

Molecules that comply with the standard physical–chemical parameters and pharmacokinetic profiles are presented in Table S6. However, one molecule was discarded for having an LD50 = 10 mg/kg (C-II) (Figure S3) and fourteen for having the probability of presenting hepatotoxicity, carcinogenicity, immunotoxicity and/or mutagenicity characteristics (Figure S4). Finally, we obtained four molecules ZINC19835187 (ZI-187), ZINC19924939 (ZI-939), ZINC19924906 (ZI-906) and ZINC70686498 (ZI-498) (Figure 2) which did not present any probability of cytotoxic characteristics, as well as the Chlorhexidine (Figure S4).

Three molecules belonging to the small library (ZI-187, ZI-939, ZI-906) were selected; molecules from this library, basically, contain drug-like properties such as molecular weight (<500 Da), hydrogen bond acceptors (HBA) (<10), hydrogen bond donors (HBD) (<5) and partition coefficient AlogP (<5). Some structural fragments of these molecules are evident, such as the case of 9h-fluorene and Piperazine (Figure 2). Thiazol was common for ZI-187 and ZI-906 (Figure 2A,C). Fluorene or 9H-fluorene is a polycyclic aromatic hydrocarbon insoluble in water and many of its derivatives have attracted wide attention as basic building blocks for the production of pharmaceuticals, drugs, lubricating materials and thermosetting plastics [51]. Piperazine, on the other hand, is an organic compound and heterocyclic amine, which has proven to be of great significance in the rational development of drugs and is found in well-known drugs with various therapeutic uses, such as antipsychotic, antihistamine, antianginal, antidepressant, anticancer, antiviral, cardio protectors, anti-inflammatory and imaging agents [52]. However, the properties of these fragments alone change considerably when they are part of other chemical molecules. Finally, the molecule ZI-498 belongs to the library of natural compounds and presents structural fragments different from the molecules previously described, ZI-498 include naphthalene, piperidine and benzene (Figure 2D).

#### 2.1.3. Molecule–Protein Interaction and Solubility Analysis

The presence of one or two hydrogen bridges was confirmed (Table S7) using the interaction complexes in the pockets established by the two predictors between the selected molecules and the 3IPK and 3QE5 proteins. This finding could indicate that the interaction between each molecules ZI-187, ZI-939 and ZI-906 with both proteins would have very stable couplings, resulting in a possible inhibition of the *S. mutans* adhesion, since it has been shown that hydrogen bridges regulate and facilitate molecular interactions [53,54,55,56,57].

**Figure 3:**
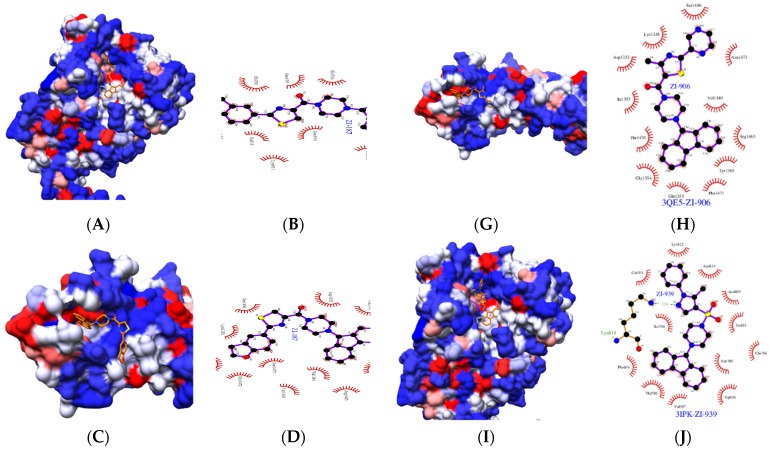

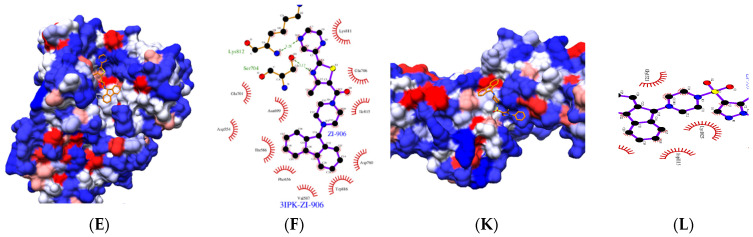
Comparison of the interactions of three molecules with A3VP1 region (PDB: 3IPK) and C-terminal region (PDB: 3QE5) from Ag I/II of *S. mutans*. Hydrophobicity in surface form is shown for the A3VP1 region interacting with (**A**) ZI-187, (**E**) ZI-906 and (**I**) ZI-939 and C-terminal region interacting with (**C**) ZI-187, (**G**) ZI-906 and (**K**) ZI-939, where the color scale corresponds to that blue is very hydrophilic and red is very hydrophobic. Molecular interactions (to 3 Angstrom radius from the molecules) between residues from A3VP1 region with (**B**) ZI-187, (**F**) ZI-906 and (**J**) ZI-939 and from C-terminal region with (**D**) ZI-187, (**H**) ZI-906 and (**L**) ZI-939.

Additionally, the hydrophobicity analysis of the two Ag I/II domains (PDB: 3IPK and PDB: 3QE5) showed that the three molecules ZI-187, ZI-906 and ZI-939 have a similar interaction with both domains, in which the interaction site is characterized by one hydrophobic and hydrophilic region (Figure 3). The 9h-fluorene fragment, which is present in the three molecules, interacts with the same residues from the PDB: 3IPK domain, Thr586-Val587-Phe656-Asp760-Trp816, which are hydrophobic, with the exception of Asp760 (Figure 3B,F,J). However, the interaction of the 9h fluorene fragment with PDB domain: 3QE5 is similar for the molecule ZI-187 and ZI-906, which interact with the residues Val1340-Gly1354-Gln1355-Arg1465-Thr1470-Phe1471 (Figure 3D,H), most of them hydrophobic. The molecule ZI-939 also shows interaction with different hydrophobic residues, but different Ile1157-Tyr1322-Ala1323 (Figure 3L). On the other hand, the fragments that are different within the three molecules interact mostly with hydrophilic residues; we found that for the PDB: 3IPK domain the molecules interact in the same way with the residues Asn699-Glu701-Ser704-Ile815, and the interactions with PDB domain: 3QE5 are also characterized by hydrophilic residues. Only the molecules ZI-187 and ZI-906 showed interactions with common residues, such as Lys1338-Asp1353-Asn1473-Ser1486 (Figure 3D,H), different from those of ZI-939, which were Lys1023-Gln1024-Leu1113-Gly1321 (Figure 3L).

Finally, a descriptive analysis about water solubility of the molecules was conducted using a consensus from 3 methods resulting in a low aqueous solubility of the molecules, but this parameter was no used as a selection criteria for molecule selection (Table S8).

### 2.2. In Vitro Assays

#### 2.2.1. Cytotoxicity and Antimicrobial Assays

**Figure 4:**
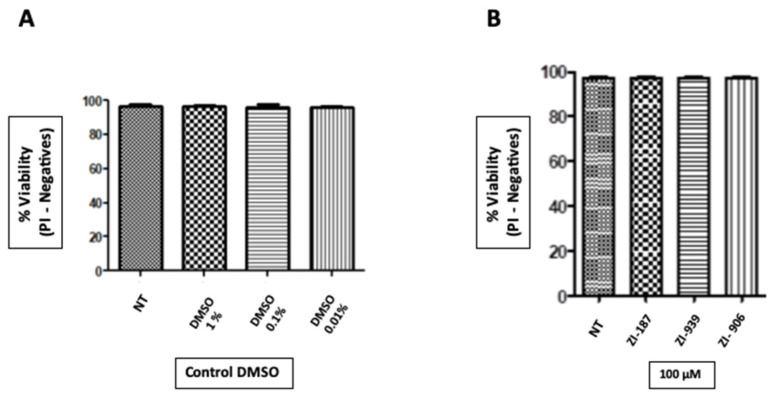
Cytotoxicity assay on periodontal ligament fibroblast cells (PLF). (**A**). Evaluation of compounds solvent cytotoxicity (DMSO 1%, 0.1% and 0.01%). NT: Not-treated. (**B**). Cytotoxicity evaluation of compounds (100 µM) ZI-187 (*p* = 0.7372), ZI-939 (*p* = 0.8) and ZI-906 (*p* = 0.7964) on PLF, by laminar flow cytometry analysis with Propidium Iodide.

It was found that molecules at concentrations of 100 µM have no effect on periodontal ligament fibroblast cells growth and the cells treated with molecules ZI-187 (*p* = 0.7372), ZI-939 (*p* = 0.8) and ZI-906 (*p* = 0.7964) (Figure 4). However, cells treated with each molecule showed changes in size and granularity. Therefore, it is important to analyze other human cell lines and add complementary analysis such as incorporation of DIOC6 for the mitochondrial membrane potential measurement [58] or apoptosis tests as Annexin V [59]. In addition, for antimicrobial assays, molecules at concentrations of 1000–100–10 µM co-cultured experiments with *S. mutans* LT-11 or *C. albicans*-NCPF 3179 did not affect their growth (*p* = <0.001) (Figures S5 and S6, respectively).

#### 2.2.2. Adhesion Assays

**Figure 5:**
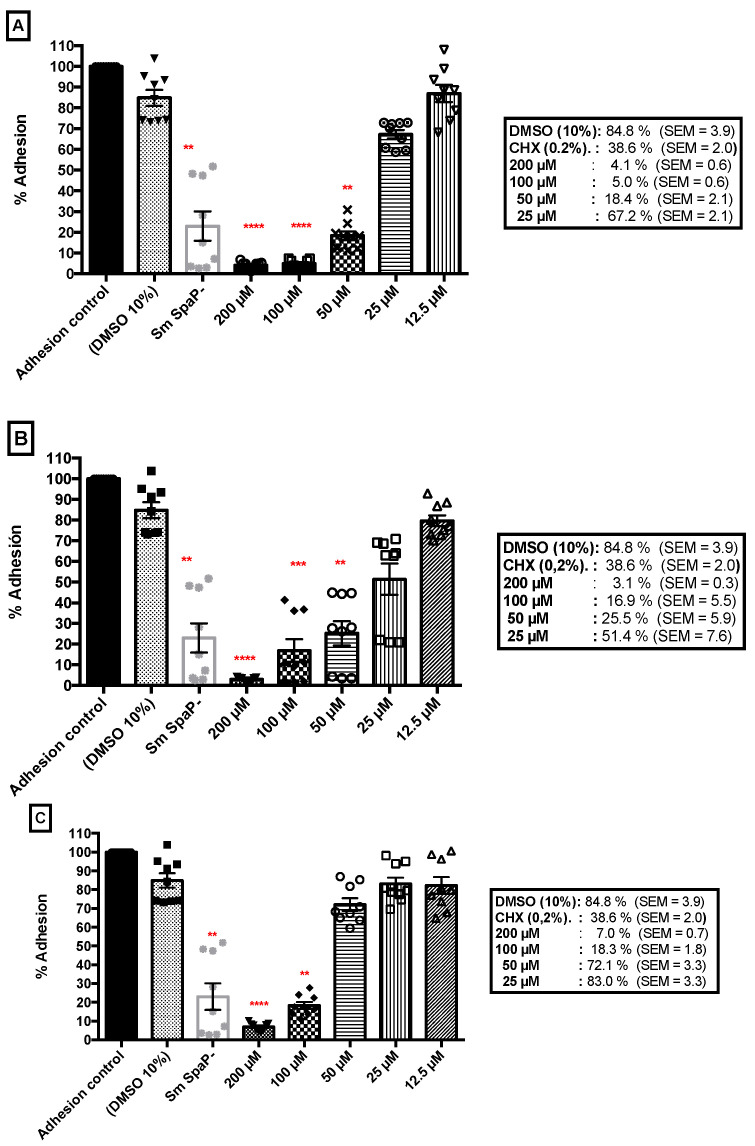
Surface adhesion of *S. mutans*-LT11 to a polystyrene microwell plate treated with molecules (**A**) ZI-187, (**B**) ZI-906 and (**C**) ZI-939. The asterisks represent the level of significance ** (*p* ≤ 0.01), *** (*p* ≤ 0.001) and **** (*p* ≤ 0.0001). All experiments were performed in triplicate and reproduced three separate times (the geometric figures above each column chart indicates each data).

The *S. mutans* LT11 three hour adhesion inhibition test with each molecule selected (ZI-187, ZI-939, ZI-906) inhibited the surface adhesion to a polystyrene microwell plate. The three molecules showed an adhesion inhibition greater than 90% at a concentration of 200 µM, 95.9% (SEM = 0.6) with ZI-187, 96.9% (SEM = 0.3) with ZI-906 and 93% (SEM = 0.7) with ZI-939 (Figure 5). This inhibition of adhesion was maintained above 90% at a concentration of 100 µm only with the molecule ZI-187 (95.0%, SEM = 0.6) (Figure 5A), and for the molecules ZI-906 and ZI-939, the inhibition capacity decreased to 83.1% (SEM = 5.5) and 81.7% (SEM = 1.8) with ZI-906 and ZI-939 (Figure 5B,C), respectively. Interestingly, the molecules ZI-187 and ZI-906 showed significant differences (*p*-Value < 0.001) in comparison to *S. mutans* Ag I/II deficient (*S. mutans* SpaP-). Up to a concentration of 50 µM, the adhesion inhibition percentages were 81.6% (SEM = 2.1) for ZI-187 and 74.5% (SEM = 5.9) for ZI-906, but there was no statistically significant difference for adhesion inhibition of 27.9% (SEM = 3.3) with ZI-939. Additionally, with these results, the IC50 (the half maximal inhibitory concentration) was calculated, finding that the IC50 for ZI-187 was 27.6 µM (95% CI = 17.4–44.5) (Figure 5A), IC50 of 28.3 µM (95% IC = 20.2–39.8) for ZI-906 and IC50 of 59.5 µM (95% CI = 37.4–95.8) for ZI-939.

These findings show the importance of the molecule fragments (9h-fluorene and Piperazine) in the adhesion inhibition, because it has been shown that bacteria have mechanisms that modify the surfaces to increase hydrophobicity and be able to adhere [60,61]. For this study, it was found that the 9h fluorene fragment is very important for adhesion inhibition of *S. mutans*, since the three molecules contain this fragment and interact with hydrophobic parts of the residues and additionally the other fragments such as 2,3 Dihydrobenzofuran in ZI-187 could be important for inhibition capacity.

**Figure 6:**
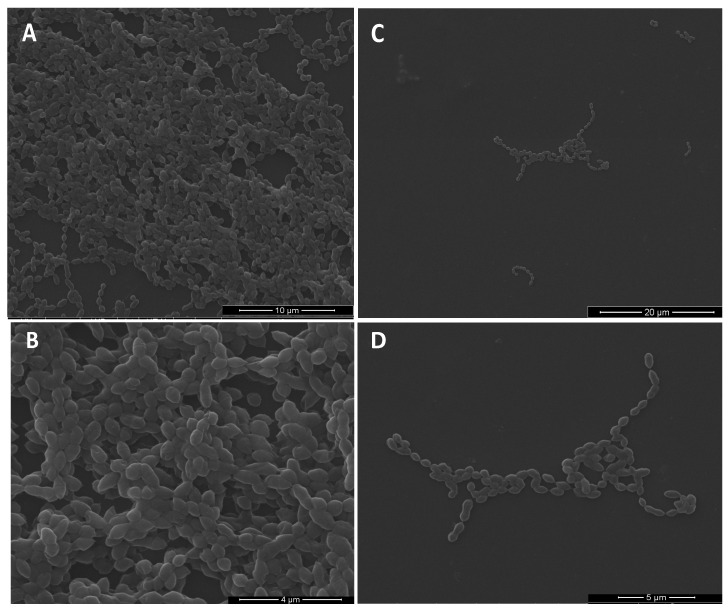
Scanning electron microscopy of *S. mutans*-LT11 surface adhesion to a polystyrene microwell plate. Without treatment at 7000× (**A**) and 19,000× (**B**); and treated with 100 µM of molecule ZI-187, at of 4000× (**C**) and 11,000× (**D**).

Additionally, an agglutination phenomenon was observed when molecules with the bacteria was mixed, only at concentrations where the inhibition was affected (Figure S7). ZI-187 was the only molecule that maintained adhesion inhibition above 90% at a concentration of 100 µM; hence, this was selected for scanning electron microscopy. Reduction in adherent bacteria was evident, and we did not observe morphological changes in *S. mutans* following treatment with ZI-187; bacteria had intact cell structure and round shapes with smooth edges (Figure 6).

There are multiple chemical strategies that could limit the development of dental biofilm; however, most of them can have side effects on teeth, soft tissues or killing oral microbiota, which show the need for specific therapies for cariogenic bacteria. Several studies have focused on blocking two important mechanisms for *S. mutans* biofilm development, such as avoiding sucrose-dependent or sucrose-independent adhesion and interference of cellular signaling “Quorum sensing” [10]. This study was carried out avoiding the sucrose-independent adhesion method, which has been aimed mainly at blocking sortase A, a transpeptidase involved at the anchoring of cell surface proteins, including Ag I/II, through the LPXTG motif. It has been found that several molecules can reduce biofilm formation, through the inhibition of the sortase A [62,63], such is the case of the natural phenol curcumin, (*Curcuma longa*) with which inhibition *S. mutans* sortase A activity with IC50: 10 µM and an MIC of 175 µM has been reported [64]. However, despite the multiple benefits of this molecule, some toxic effects have also been evidenced related to the high doses as a result of its use as a supplement in the diet [40,41]. Another natural product is named Morin; it has an inhibitory effect against *S. mutans* SrtA with IC50: 27.2 µM [65,66]. Morin, another natural product, has an inhibitory effect against *S. mutans* SrtA with IC50: 27.2 µM [67], similar to the IC50 obtained with our molecules ZI-187 and ZI-939 (IC50: 27.6 y 28.3 µM, respectively). However, the antimicrobial activity of Morin has also been reported against *S. mutans* [68], differently from the molecules found in this study that did not present cytotoxic or antimicrobial activities.

#### 2.2.3. Molecular Dynamics Simulations (MD)

**Figure 7:**
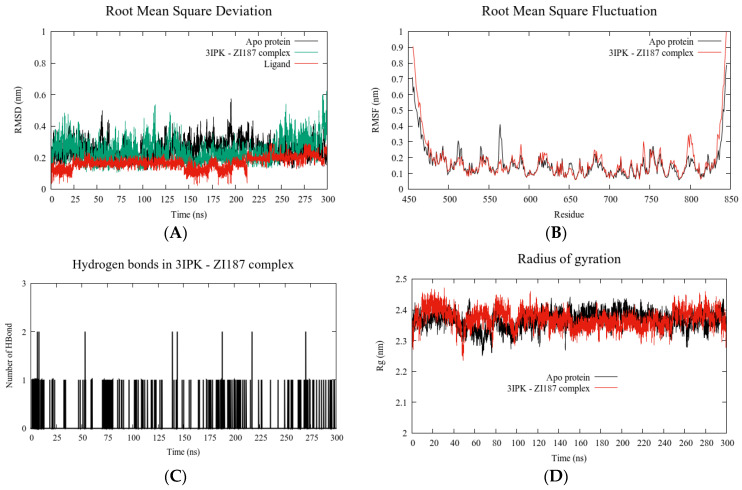
Molecular dynamics simulations analysis. Root Mean Square Deviation (RMSD) (**A**), Root Mean Square Fluctuation (RMSF) (**B**), hydrogen bonds (**C**) and Radius of gyration (**D**) calculated for ZI-187/3IPK complex.

Using molecular dynamics simulation, we found that the complex 3IPK/ZI-187 attained a high stability after 300 ns; during this time, the 3IPK protein did not have strong changes when it was coupled with ZI-187 (Figure 7A), which could indicate that the interacting molecule moves on the pocket, but not drastically; this means that it does not destabilize the complex; in agreement with the RMSD result, no significant fluctuation of amino acid residues was observed; however, between residues 550–600 and 800–850, some differences were found between the APO protein for 3IPK (black) and the complex (red) (Figure 7B), which could be due to the specific 3IPK residues that interact with ZI-187, since different types of interactions of ZI-187 with residues Leu 553-Asp 554-Thr 586-Val 587 and Lys 811-Lys 812-Asn 814-Ile 815-Trp 816 were identified (Figure S6). In addition, 1 and 2 H-bonds were observed between the complex during the simulation time (Figure 7C) and a constant protein structure compactness in the complex (Figure 7D); these two parameters would support the stability of the interaction between ZI-187 and 3IPK, giving a possible explanation for the molecule’s ability to inhibit the adhesion of *S. mutans*.

Finally, the present work allowed a virtual search strategy and selection pipeline for adhesion inhibitory molecules of a cariogenic bacteria but that could be applicable to any pathogen to be established. This study, to the best of our knowledge, is the first report that uses the *S. mutans* Ag I/II as a target, since previous studies were mainly performed on Gtfs or sortase A proteins. Three molecules were selected—ZI-187, ZI-939 and ZI-906—without showing any cytotoxic effect on periodontal ligament fibroblasts or antimicrobial activity on *S. mutans* or *C. albicans*. However, it is suggested to perform assays in other different human cell lines, as well as on other microorganisms of oral cavity importance to evaluate in a much wider range other possible effects. It was also found, as expected, that the molecules selected had a significant effect in terms of reduction in the *S. mutans* surface adhesion as a single microorganism, but it is very important to carry out complementary studies on multispecies biofilms models, also to identify through transcriptomic analysis if there are variations in the expression of adhesion genes dependent and independent of sucrose when treating bacteria with the selected molecules, as well as genes that participate in cell signaling during the biofilm development process. Similarly, an in vivo cariogenic model should be established in order to insight the anticariogenic capacity of these molecules.

## 3. Materials and Methods

### 3.1. Structure-Based Virtual Screening

#### 3.1.1. Target Proteins Selection

Three-dimensional protein structures for the virtual search were selected using the Ag I/II of *S. mutans* sequence AFR75221.1 (NCBI https://www.ncbi.nlm.nih.gov/) and the 3D SWISS-MODEL software (http://swissmodel.expasy.org/). Three-dimensional structures of two Ag I/II protein fragments, 3IPK (PDB-ID) [21] and 3QE5 [20], were used for a sequence and protein structure similarity analysis using a Protein Basic Local Alignment Search Tool-NCBI (BLAST-P) and the Flexible structure Alignment by Chaining Aligned fragment pairs allowing Twists (FAT-CAT) server [69] to search for similar (rigid) protein structures, using similarities only with a *p*-value < 0.05. Subsequently, for 3IPK and 3QE5 proteins, an analysis of binding sites “Pockets” using meta-servers MetaPocket 2.0 [49] and COACH [50] was carried out. Three-dimensional structures of the proteins were obtained in PDB format and edited in AutoDockTools 4.0 (http://mgltools.scripps.edu) [70]. Molecular docking of molecules with AgI/II protein fragments was performed at the Texas Advanced Computing Center (TACC: Texas Advanced Computing Center; Austin, TX, USA) using 3 libraries—“ZINC (Lrg)” of ~642,759, Library “ZINC (Sm)” of ~46,702 molecules and “ZINC Natural Cmpds (Large)” of 194,090 from the ZINC15 database [71], a total of ~883,551 molecules.

#### 3.1.2. Molecule Selection

Two methodologies were used for molecule selection—one according to the molecular docking score and another according to the number of pockets in which molecules interacted—classification of molecules that interacted in most pockets was carried out using a script executed in the R-studio package (Version 1.0.156); ten molecules were arbitrarily selected from each methodology. An in silico analysis was performed using the QuikProp application (Version 3.2) from Schrödinger software (Schrödinger Release 2020-4: QikProp, Schrödinger, LLC, New York, NY, USA, 2020.), according to [72] with some modifications, in order to analyze pharmacokinetic profiles such as absorption, distribution, metabolism and excretion (ADME). Molecules that had more than two violations of the Lipinski’s Rules and that did not comply with more than two of the standard physical–chemical parameters established by 95% of the known drugs, according to Schrödinger’s QuikProp program repositories, were excluded.

For computational toxicity prediction of the molecules, the Protox-II program was used [73], for acute toxicity, hepatotoxicity, cytotoxicity, carcinogenicity, mutagenicity and immunotoxicity. In addition, the molecules lethal dose 50 (LD50) (mg/kg) was calculated and classified according to the Globally Harmonized System of Classification and Labeling of Chemicals (GHS). For this analysis, chlorhexidine was used as the reference drug, one of the most commonly prescribed antiseptic agents in dentistry; the experimental LD50 values of oral administration in mice of this drug were taken from the Pfizer chlorhexidine technical data sheet [74]. Molecules above class 3 and without any probability of toxicity were selected.

#### 3.1.3. Molecule–Protein Interaction and Solubility Analysis

ZINC19835187 (Database code Zinc15), ZINC19924906 and ZINC19924939, (ZI-187, ZI-906 and ZI-939, respectively) were selected, and the H-bonding and hydrophobicity interactions between the molecules and the two protein fragments 3ipk–3qe5 were identified [75] using Biovia Discovery Studio [76] and Chimera [77] softwares. The swissADME web server (http://www.swissadme.ch/) was used to predict water solubility characteristics of the molecules, using a consensus of three methods Log S (ESOL), Log S (Ali) and Log S (SILICOS-IT) [78].

### 3.2. In Vitro Assays

Three molecules were purchased (ZI-498 was not available for sale) from the MolPort company (https://www.molport.com). Stock solution of the molecules were diluted in 100% dimethyl sulfoxide (DMSO) at a concentration of 10^4^ µM (ZI-187 (MW = 479.6) = 4796 µg/mL; ZI-906 (MW = 453.6) = 4535, 6 µg/mL; ZI-939 (MW = 470.6) = 4075.9 µg/mL).

#### 3.2.1. Cytotoxicity and Antimicrobial Assays

Cytotoxic effects of ZI-187, ZI-906 and ZI-939 on periodontal ligament fibroblast cells (FLP) treated for 24 h with each molecule were evaluated and analyzed by flow cytometry with propidium iodide (PI), using 100 µM of each molecule, and DMSO 1%. *S. mutans*-Lt11 (UB579 WT) [79] and *C. albicans* NCPF 3179 (NCPF, 1986) were cultured overnight in broth BHI BD^®^ at 37 °C shaking at 250 RPM. The next day, *S. mutans* and *C. albicans* suspension in BHI broth (180 µL/well, OD_600 nm_ = 0.1) was seeded into 96-well plates (Costar, Cambridge, MA, USA) with 20 µL of the molecules (concentrations of 1.000, 100, 10 µM. DMSO 10%), as a negative control (death) 0.2% Chlorhexidine digluconate (Farpag^®^) was used, while the corresponding broth without molecules was used as a positive growth control, as well as those treated with DMSO 10% [80]. After incubation for 24 h at 37 °C shaking at 250 RPM, the absorbance was measured in an Epoch™ Microplate spectrophotometer (BioTek^®^) (OD_600 nm_), to evaluate cell growth and to establish the minimum inhibitory concentration (MIC).

#### 3.2.2. Adhesion Assay

*S. mutans-Lt11 (UB579 WT)* was cultured in BHI broth (BD^®^) overnight at 37 °C shaking at 250 RPMI. The culture medium was discarded, and bacteria were washed with phosphate buffer solution (PBS 1X) by centrifugation at 3000 RPM for 10 min. Subsequently, bacterial suspension in PBS 1X, OD_600 nm_ = 1 was measured using an Epoch™ Microplate spectrophotometer (BioTek^®^) and 180 µL were inoculated into a 96-well microplate (NEST^®^, Ref: 701001) with 20 µL of each molecule at 200, 100, 50, 25 and 12.5 µM and incubated for three hours at 37 °C shaking at 250 RPM [81]. *S. mutans* SpaP-strain (mutant for AgI/II also called SpaP) and *S. mutans*-Lt11 treated with DMSO 10% were used as a control. Plate wells were washed with water and adherent cells were stained by adding 200 μL 0.05% crystal violet for 15 min, washed and measured by absorbance at 600 nm after addition of 30% glacial acetic acid.

#### 3.2.3. Data Analysis

All experiments were performed in triplicate and reproduced three separate times. Cell viability percentages were reported as negative % PI ± SEM and analyzed by one-way ANOVA, followed by a Dunnett’s multiple comparison test. In *S. mutans* and *C. albicans* growth and adhesion inhibition analysis, the OD data were normalized to percentages and analyzed using D’Agostino and Pearson omnibus and Shapiro–Wilk normality test; subsequently, the nonparametric Kruskal–Wallis test with Dunnett’s multiple comparisons were carried out against the controls *S. mutans* and *C. albicans* treated with DMSO 10%. Finally, the half maximal inhibitory concentration (IC50) was calculated. GraphPad Prism version 6.0 (GraphPad Software, La Jolla, CA, USA, www.graphpad.com) was used, and values of *p* < 0.05 were considered statistically significant.

#### 3.2.4. Scanning Electron Microscopy (SEM)

Surface adhesion assays were performed on Thermo Scientific Nunc Lab-Tek and Lab Tek II Chamber Slides, using *S. mutans*-Lt11 untreated and treated with molecule ZI-187 (100 µM). After incubation for three hours at 37 °C shaking at 250 RPM, each sample was washed three times with PBS 1X, then the samples were fixed with 2.5% glutaraldehyde (0.1 M PBS) for 24 h at 4 °C. Finally, the slides were washed three times with distilled water and dehydrated by immersion in solutions of ascending concentrations of ethanol 70, 90 and 100% (10 min each) and dried overnight in a laminar flow cabinet. The samples were covered with gold and visualized using a FEI QUANTA-200TM scanning electron microscope with a variable range acceleration voltage of 1–30 KV.

#### 3.2.5. Molecular Dynamics Simulations (MD)

MD simulations were carried out using the GROMACS 4.5.5 package [82]. Molecule ZI-187 was docked to the 3IPK protein pocket with the highest binding affinity (P1 by COACH predictor). The ZI-187/3IPK complex and the 3IPK protein in its APO state (reference state) were used as initial coordinates for MD simulations. Finally, both systems were subjected to a 300 ns production stage, using a 2 fs time step. The equilibrations and productions were carried out using a temperature of 310 K (36.85 °C) and a 1 bar pressure. Descriptors such as the root of the mean square deviation (RMSD), the root of the mean square fluctuation (RMSF) and hydrogen bonds present in the protein–ligand complex were followed with the tools contained g rms, g rmsf and g hbond, respectively.

## Figures and Tables

**Table 1:** Energy interaction data obtained from recoupling using Autodock Vina with an exhaustiveness of twenty for the 3IPK and 3QE5 proteins with the selected molecules, molecule libraries and number of pockets in which each molecule interacted. The molecule highlighted in black was the only one that interacted with high affinity in different sites in both 3IPK and 3QE5 proteins. COA: COACH program. MET: Metapocket 2.0 program. Values highlighted in light blue represent the lowest interaction energy values.

Number of Molecules Interaction Pockets	Molecules	Library	3IPK						3QE5					
			P1		P2		P3		P1		P2		P3	
			COACH	MET	COACH	MET	COACH	MET	COACH	MET	COACH	MET	COACH	MET
Not applicable	ZINC68568370	NAT	−12.8	−12.8	−7.5	−9	−9.4	−10.9	−8.5	−8.8	−8.1	−8.9	−7.1	−7.7
	ZINC70669788	NAT	−11.4	−12.8	−7.5	−8.4	−8.7	−10.1	−7.9	−8.7	−7.7	−7.6	−7.6	−6.5
	ZINC70669789	NAT	−11.5	−12.7	−7.3	−8.3	−8.8	−9.8	−8.1	−9	−7.9	−7.8	−7.8	−6.3
	ZINC34257514	NAT	−12.6	−12.6	−6.7	−8.8	−7.7	−9.8	−6.9	−8.7	−6.8	−7.6	−6.7	−7.6
	ZINC04817561	NAT	−10.3	−12.4	−10.1	−8.3	−8.6	−12.4	−7.2	−9.2	−7.1	−7.9	−7.2	−7
	ZINC67912808	NAT	−12.3	−12.5	−8.2	−8.8	−8.4	−9.1	−7.6	−9.5	−7.6	−8.1	−7.1	−6.8
	ZINC70686498	NAT	−12.2	−12.2	−7.8	−8.2	−8	−9.9	−7.4	−8.1	−7.3	−9.6	−6.6	−7.1
	ZINC04015296	NAT	−11.7	−11.6	−8.4	−9.8	−9	−11.7	−9.3	−11.4	−7.5	−9.3	−7.4	−9.8
	ZINC08594547	LRG	−11.6	−11.7	−7.7	−8.5	−8.1	−11.4	−7.6	−8.6	−7.1	−8.2	−6.5	−8.3
12	ZINC00970517	SM	−9.4	−9.4	−9	−8.2	−7	−9	−6.7	−7.7	−6.3	−7.5	−5.9	−7.3
12	ZINC01033612	SM	−9.3	−9.5	−8.7	−7.7	−7.3	−9.4	−7.4	−7.8	−7	−8.1	−7.4	−7.5
12	ZINC08647964	SM	−9.7	−9.8	−9.4	−7.9	−7.4	−10.4	−6.9	−8.4	−6.8	−8.7	−6.2	−7.5
12	ZINC12369546	SM	−9.5	−10	−9.1	−8.3	−7.5	−9.8	−7.9	−8.3	−6.7	−7.8	−6.8	−8.5
12	ZINC19924906	SM	−11.1	−11	−7.2	−8.2	−7.1	−9.2	−6.9	−8.3	−6.7	−7.5	−6.8	−7.5
12	ZINC03120327	SM	−9.6	−9.7	−10.1	−7.9	−7.1	−10.1	−6.7	−8	−7.1	−8	−6.9	−8.5
12	ZINC19835160	SM	−10.3	−9.5	−6.9	−8.7	−7.7	−9.7	−6.6	−8.4	−6.7	−8	−5.8	−6.9
12	ZINC19835187	SM	−10.7	−10.7	−9.2	−8.8	−8.5	−10.3	−8.1	−8.7	−7.5	−8.4	−6.9	−8.8
12	ZINC19924939	SM	−10.4	−10.4	−9	−8.9	−7.2	−8.1	−7.6	−7.5	−7.1	−8	−6.4	−7.9
12	ZINC59608258	SM	−9.4	−10.1	−7.8	−7.9	−7.2	−8.9	−6.8	−7.7	−6.1	−7.6	−6.3	−6.7

## Supplementary Materials

The following are available online at https://www.mdpi.com/1422-0067/22/1/377/s1.
