# Supplementary material for: In Silico Selection and In Vitro Evaluation of New Molecules That Inhibit the Adhesion of Streptococcus mutans through Antigen I/II
Source: Int J Mol Sci. 2020 Dec 31;22(1):377. doi: 10.3390/ijms22010377 (PMC7795114; doi:10.3390/ijms22010377)
Supplement: Supplementary file 1 [file ijms-22-00377-s001.pdf]

International Journal of
Molecular Sciences

MDPI

Article

In silico selection and *in vitro* evaluation of novels inhibitors of *Streptococcus mutans* Ag I/II surface protein

Raúl E. Rivera-Quiroga[1,2*], Néstor Cardona[1], Leonardo Padilla[2], Wbeimar Rivera[3], Cristian Rocha-Roa[4], Mayri A. Diaz De Rienzo [5], Sandra M. Morales[3], María C. Martinez[3]

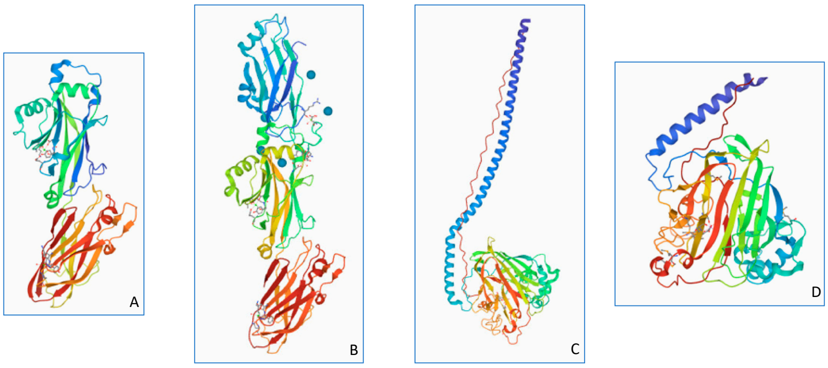

Figure S1. Crystals structures of proteins obtained from the Protein Data Bank (PDB), associated with *S. mutans* Ag I/II: Crystals Structures of 3OPU (A) and 3QE5 (B) from the C-terminal region and crystals structures of 3IPK (C) and 3JMM (D) from the A3VP1 region of *S. mutans* Ag I/II.

A

PREDICTOR	PROTEIN FRAGMENT	POCKET	COORDINATES			Z-SCORE
			X	Y	Z	
MetaPocket	3IPK	1	7.739	37.581	26.828	16.97
		2	26.182	63.410	1.527	4.35
		3	8.756	41.334	17.724	1.63

B

PREDICTOR	PROTEIN FRAGMENT	POCKET	COORDINATES			C-SCORE
			X	Y	Z	
COACH	3IPK	1	8.442	42.402	30.522	0.375
		2	16.431	32.680	14.360	0.051
		3	-5.605	37.483	28.032	0.051

C

PREDICTOR	PROTEIN FRAGMENT	POCKET	COORDINATES			Z-SCORE
			X	Y	Z	
MetaPocket	3QE5	1	26.775	35.391	81.221	13.69
		2	35.450	42.813	114.619	5.86
		3	101.525	56.924	161.536	5.64

D

PREDICTOR	PROTEIN FRAGMENT	POCKET	COORDINATES			C-SCORE
			X	Y	Z	
COACH	3QE5	1	69.690	53.887	128.439	0.21
		2	55.817	51.819	112.584	0.20
		3	72.663	44.614	148.421	0.19

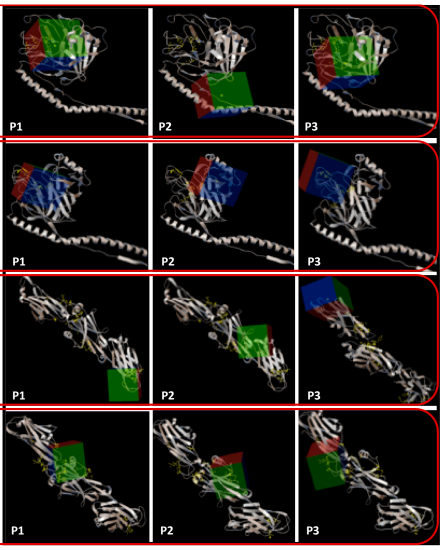

Figure S2. Scheme for description of coordinate data and location of the binding sites for the search compounds with affinity to the 3IPK and 3QE5 proteins, established by the Metapocket and COACH programs. Coordinate data in the X, Y and Z axes for 3 ligand binding sites delimited in the three images (P1, P2, P3) of the protein structure, with the green, blue and red color box (Prepared in AutoDockTools); established for 3IPK by the Metapocket (A) and COACH (B) program and for 3QE5 by Metapocket (C) and COACH (D).

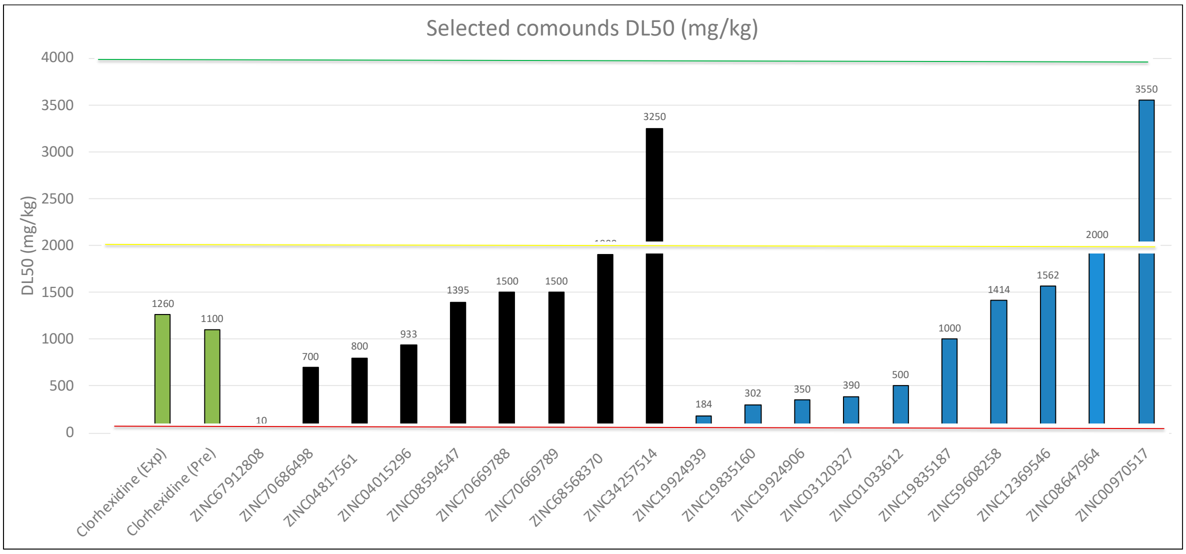

Figure S3. Ld50 (mg / kg) data calculated for selected compounds. Chlorhexidine was used as a reference and its experimental and predictive LD50 values 1260 and 1100 (mg / kg) respectively, are included (green bars). The red line represents the LD50 reference point, based on the Chlorhexide experimental LD50 data. The black bars represent the compounds that were selected according to the values of interaction energies and the blue bars represent the compounds selected according to the number of sites of interaction in which it is coupled. According to the LD50, the red line establishes the limit of the compounds classified as probably fatal (Groups I and II), the yellow line for probably toxic compounds (Groups III and IV) and the green line probably harmful or non-toxic (Groups V and VI).

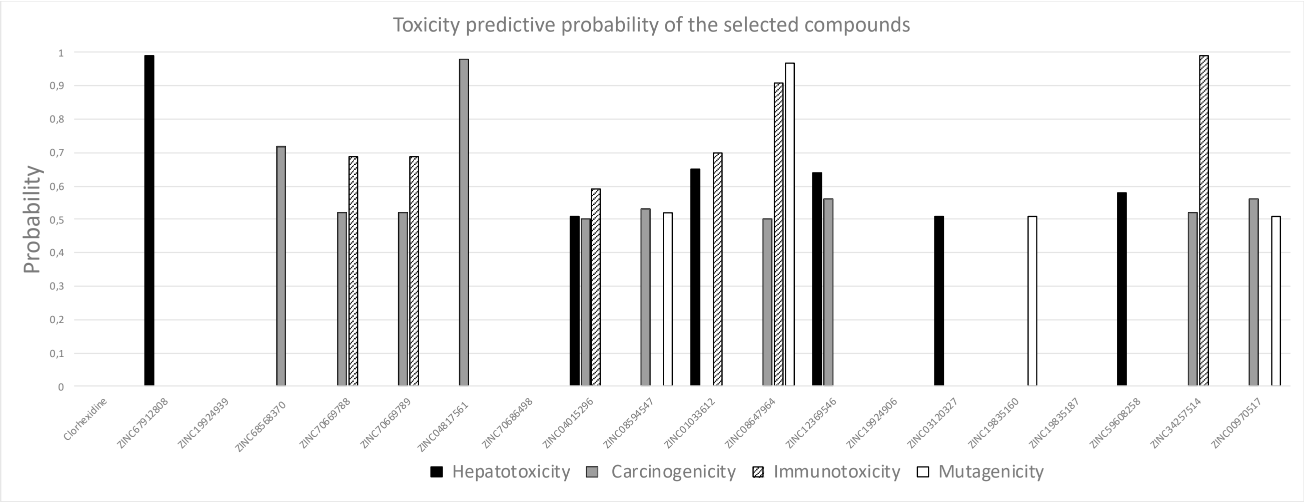

Figure S4. Toxicity predictive probability results (Hepatotoxicity - Carcinogenicity - Immunotoxicity - Mutagenicity) of the selected compounds, calculated in the ProTox-II virtual laboratory. Chlorhexidine was included in this analysis as a reference, making the same calculation.

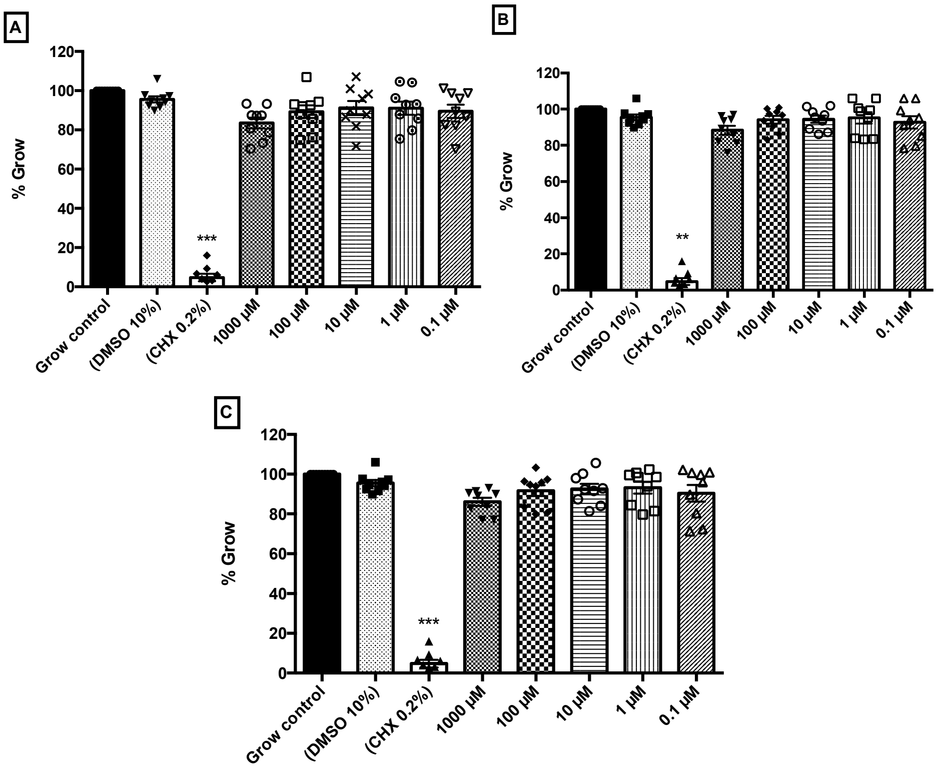

Figure S5. Cytotoxicity evaluation of compounds ZI-187, ZI-939 and ZI-906 at concentrations of 1000 -100 - 10 - 1- 0.1 μM, on the *S. mutans*-LT11 growth treated for 24 hours. Growth control (*S. mutans* - LT11), vehicle control (*S. mutans* - LT11 in DMSO 10%), death control (CHX: Chlorhexidine 0.2%). For this test the DO (100%): 2.478 (ES = 0.19) and P-value = <0.001.

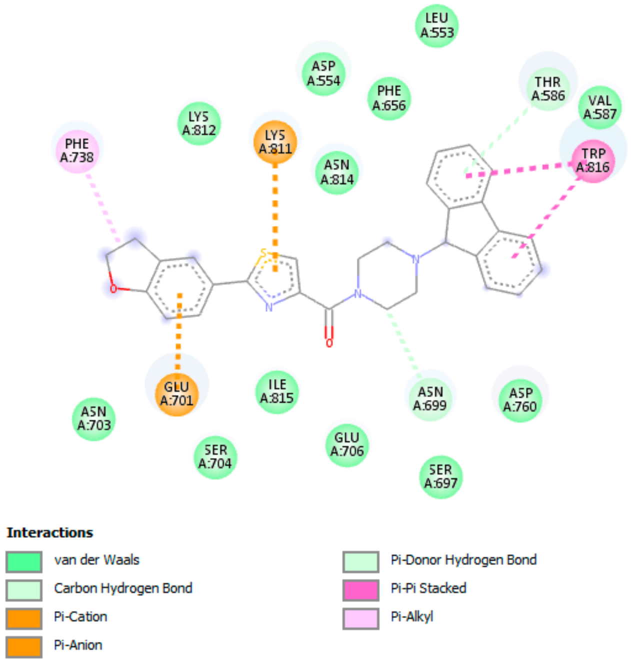

Figure S6. Analysis of the interactions found between compound ZI-187 and pocket 1 of the 3IPK protein.

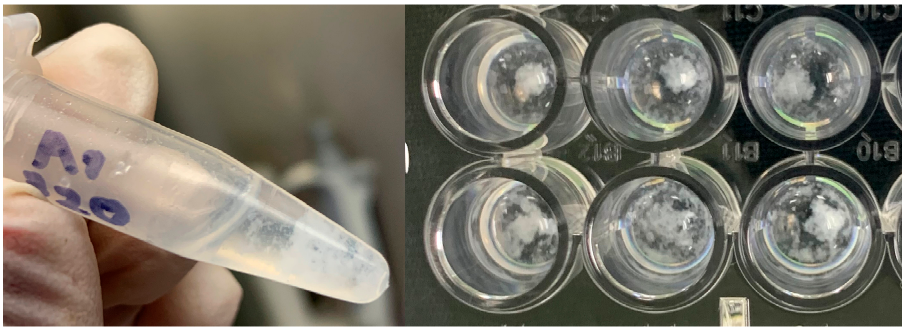

Figure S7: Photographic of the agglutination generated to the *S.mutans* - LT11 by adding any molecules ZI-187, ZI-939 or ZI-906 just in the concentrations where adhesion inhibition occurred.

Table S1: Results obtained from crystals structures proteins, using as template the *S. mutans* Ag I/II sequence, in the Swiss-model program (http://swissmodel.expasy.org/).

Model	Template	GMQE	QMEAN	Seq - Similarity	Range aa (total)	Coverage	Seq - Identity (%)
1	3ipk.1.A	0.22	0.87	0.59	392 – 870 (479)	0.31	94.77
2	3qe5.1.A	0.22	0.44	0.60	996 – 1482 (487)	0.32	98.38
3	1jmm.1.A	0.15	-0.24	0.58	464 – 823 (360)	0.23	93.04
4	3opu.1.A	0.12	0.06	0.60	1152 – 1489 (337)	0.22	97.05

Table S2. Amino acid sequences description obtained from significant alignments with 3IPK and 3QE5, using BLAST-P (NCBI) limited to records that include human sequences (taxid: 9605).

AgI/II fragment	Protein	Description	Max Score	Total Score	Query Cover	E value	Per. Ident	Accession
3IPK (A3VP1 Region)	zonadhesin isoform 3 precursor	*Homo sapiens*	43.9	43.9	16%	0.003	36.26%	NP_003377.2
	zonadhesin splice variant 6	*Homo sapiens*	43.9	43.9	16%	0.003	36.26%	AAL04413.1
	zonadhesin splice variant 5	*Homo sapiens*	43.9	43.9	16%	0.003	36.26%	AAL04412.1
	zonadhesin variant 1	*Homo sapiens*	43.9	43.9	16%	0.003	36.26%	AAK01431.1
	zonadhesin variant 4	*Homo sapiens*	43.9	43.9	16%	0.003	36.26%	AAK01434.1

	zonadhesin variant 2	*Homo sapiens*	43.9	43.9	16%	0.003	36.26%	AAK01432.1
3QE5 (C- Terminal Region)	Chain A, The Crystal Structure of E2-25k And Ubiquitin Complex	*Homo sapiens*	36.6	36.6	15%	0.25	31.03%	3K9P_A
	Chain B, REST COREPRESSOR 1	*Homo sapiens*	32.7	32.7	5%	4.4	58.62%	2IW5_B
	Chain A, Transitional Endoplasmic Reticulum Atpase	*Homo sapiens*	31.6	31.6	5%	9.1	51.72%	4KDI_A

Table S3. Amino acid sequences description obtained from significant alignments with 3IPK and 3QE5, using BLAST-P (NCBI) limited to records that include: Procaryotae (taxid: 2) and exclude: humans (taxid: 9605) and *S. mutans* (taxid: 1309). The name of the bacteria highlighted in red, represents those that are found in only one of the 3IPK or 3QE5 fragments.

**AgI/II fragment**	**Protein**	**Description**	**Max Score**	**Total Score**	**Query Cover**	**E value**	**Per. Ident**	**Accession**
3IPK (A3VP1 Region)	agglutinin receptor SSP-5	*Streptococcus troglodytae*	978	1200	98%	0.0	96.93%	BAQ24775.1
	agglutinin receptor	*Streptococcus anginosus*	882	882	98%	0.0	88.75%	PRT64203.1
	antigen I/II family LPXTG-anchored adhesin	*Streptococcus intermedius*	837	837	96%	0.0	85.00%	WP_125364452.1
	antigen I/II family LPXTG-anchored adhesin	*Streptococcus gordonii*	828	981	98%	0.0	81.87%	WP_045772712.1
	antigen I/II family LPXTG-anchored adhesin	*Streptococcus parasanguinis*	828	1093	98%	0.0	82.21%	WP_061590679.1
	antigen I/II family LPXTG-anchored adhesin	*Streptococcus macacae*	825	919	99%	0.0	84.24%	WP_003079727.1
	antigen I/II family LPXTG-anchored adhesin	*Streptococcus orisratti*	792	1236	99%	0.0	79.80%	WP_018375205.1

	antigen I/II family LPXTG-anchored adhesin	*Streptococcus oralis*	787	1048	98%	0.0	78.21%	WP_045593440.1
	protein I/II V-region	*Streptococcus intermedius*	786	786	77%	0.0	99.22%	AAF20184.1
	protein I/II V-region	*Streptococcus constellatus*	784	784	77%	0.0	98.97%	AAF20183.1
	antigen I/II family LPXTG-anchored adhesin	*Streptococcus sanguinis*	774	1039	98%	0.0	77.35%	WP_004193814.1
	LPXTG cell wall anchor domain-containing protein	*Granulicatella adiacens*	769	819	98%	0.0	76.37%	WP_005605884.1
	antigen I/II family LPXTG-anchored adhesin	*Streptococcus sobrinus*	766	1006	98%	0.0	76.83%	WP_019784233.1
	antigen I/II family LPXTG-anchored adhesin	*Streptococcus downei*	761	1002	98%	0.0	76.63%	WP_002996622.1
3QE5 (C- Terminal Region)	antigen I/II family LPXTG-anchored adhesin	*Streptococcus intermedius*	932	932	96%	0.0	93.35%	WP_117283309.1
	antigen I/II family LPXTG-anchored adhesin	*Streptococcus anginosus*	927	927	96%	0.0	92.14%	WP_143969960.1
	antigen I/II family LPXTG-anchored adhesin	*Streptococcus macacae*	885	885	96%	0.0	87.90%	WP_003079727.1
	antigen I/II family LPXTG-anchored adhesin	*Streptococcus orisratti*	816	816	96%	0.0	81.85%	WP_018375205.1
	antigen I/II family LPXTG-anchored adhesin	*Streptococcus sobrinus*	812	812	96%	0.0	76.81%	WP_019771067.1
	antigen I/II family LPXTG-anchored adhesin	*Streptococcus downei*	808	808	96%	0.0	76.81%	WP_002996622.1
	antigen I/II family LPXTG-anchored adhesin	*Streptococcus criceti*	798	798	96%	0.0	76.21%	WP_004225946.1
	LPXTG cell wall anchor domain-containing protein	*Granulicatella adiacens*	764	764	96%	0.0	78.23%	WP_005605884.1

	antigen I/II family LPXTG-anchored adhesin	*Streptococcus sanguinis*	743	743	96%	0.0	70.77%	WP_125377155.1

Table S4. Amino acid sequences description obtained from significant alignments with 3IPK and 3QE5, using BLAST-P (HOMD) limited to oral bacterial. The name of the bacteria highlighted in red, represents those that are found in only one of the 3IPK or 3QE5 fragments.

**AgI/II fragment**	**Protein**	**Description**	**Max Score**	**Per. Ident**	**E value**
3IPK (A3VP1 Region)	Cell surface antigen I/II	*Streptococcus mutans*	838	86	0.0
	Major cell-surface adhesin PAc	*Streptococcus intermedius*	770	83	0.0
	Agglutinin receptor	*Streptococcus oralis*	682	78	0.0
	Major cell-surface adhesin Pac	*Streptococcus downei*	665	78	0.0
	Agglutinin receptor	*Streptococcus sanguinis*	659	77	0.0
	Major cell-surface adhesin Pac	*Granulicatella adiacens*	658	76	0.0
	Agglutinin receptor	*Streptococcus gordonii*	644	76	0.0
	Major cell-surface adhesin Pac	*Parvimonas micra*	451	68	1E-125
	Agglutinin receptor	*Cryptobacterium curtum*	165	45	5E-39
	Agglutinin receptor	*Mogibacterium timidum*	154	46	6E-36
	Agglutinin receptor	*Atopobium parvulum*	150	43	1E-34
	Agglutinin receptor	*Abiotrophia defectiva*	147	42	8E-34
3QE5 (C- Terminal Region)	Major cell-surface adhesin Pac	*Streptococcus mutans*	862	88	0.0
	Major cell-surface adhesin Pac	*Streptococcus intermedius*	816	86	0.0
	Cell surface antigen I/II	*Streptococcus anginosus*	750	83	0.0

	Major cell-surface adhesin Pac	*Granulicatella adiacens*	689	77	0.0
	Major cell-surface adhesin Pac	*Streptococcus downei*	688	77	0.0
	Major cell-surface adhesin PAc	*Streptococcus sobrinus*	688	78	0.0
	Agglutinin receptor	*Streptococcus sanguinis*	629	74	1E-178
	Agglutinin receptor	*Streptococcus gordonii*	624	73	1E-177
	Cell surface antigen I/II	*Streptococcus oralis*	622	73	1E-177
	Cell surface antigen I/II	*Streptococcus sinensis*	333	52	8E-90
	Agglutinin receptor	*Streptococcus agalactiae*	329	61	2E-88
	Agglutinin receptor	*Streptococcus vestibularis*	327	60	7E-88

Table S5. Structural homologies result for the 3IPK and 3QE5 search with the FATCAT program. The data correspond only to results with a P-value <0.05.

Query	structure	length	score	P-value	twist	opt-len	opt-rmsd	chain-rmsd	align-len	gap	seq-ide(%)
3IPK	3ipkA	489	1464.00	0.00e+00	0	489	0.00	0.00	489	0	100.00
	3ioxA	489	1450.65	0.00e+00	0	489	0.64	0.63	489	0	100.00
	3ipkB	489	1448.24	0.00e+00	0	489	1.60	1.60	489	0	100.00
3QE5	3qe5A	487	1440.00	0.00e+00	0	487	0.00	0.00	487	0	100.00
	3qe5B	487	1436.40	0.00e+00	0	487	0.59	0.57	487	0	100.00
	4tshB	485	1358.85	0.00e+00	0	464	0.64	0.64	486	22	95.47

Table S6: Physicochemical parameters and predictive pharmacokinetic profiles result of the selected compounds. Calculated in the QuikProp application version 3.2 of the Schrödinger software. Pka (Calculated in ChemAxon's Marvin software).

	Molecular Weight	Total SASA	No. of Rotatable Bonds	as Donor - Hydrogen Bonds	as Acceptor - Hydrogen Bonds	QP log P for octanol/ water	Apparent Caco-2 Permeability (nm/sec)	Apparent MDCK Permeability (nm/sec)	QP log Kp for skin permeability	Lipinski Rule of 5 Violations	% Human Oral Absorption in GI (+-20%)	Qual. Model for Human Oral Absorption	Pka
COD	(130.0 / 725.0)	(300.0 /1000.0)	(0.0 /15.0)	(0.0/6.0)	(2.0 /20.0)	(-2.0 / 6.5)	(<25 poor, >500 great)	(<25 poor, >500 great)	(Kp in cm/hr)	(maximum is 4)	(<25% is poor)	(>80% is high)	
ZINC68568370	504,6	790,9	10,0	0,0	0,0	11,4	9906,0	5899,0	2,2	2,0	100,0	low	No ionizable group
ZINC70669788	605,0	935,2	4,0	2,0	5,4	7,9	1180,0	591,0	-2931,0	2,0	100,0	low	(-0,58) - (-1,19)
ZINC70669789	605,0	925,7	4,0	2,0	5,4	7,9	1256,0	633,0	-2878,0	2,0	100,0	low	(-0,58) - (-1,19)
ZINC34257514	509,6	807,8	3,0	0,0	4,5	6,4	564,0	266,0	-3,0	2,0	88,0	low	No ionizable group
ZINC04817561	522,6	788,6	2,0	0,0	7,0	5,4	2447,0	1301,0	-2,2	2,0	93,0	low	(9,32) -(3,93) - (3,32)
ZINC67912808	634,2	767,7	2,0	3,0	8,0	5,8	3052,0	3542,0	-2035,0	2,0	100,0	low	(15,05) - (13,69) - (13,69)
ZINC70686498	554,7	873,0	2,0	1,0	8,0	4,7	203,0	247,0	-2,7	1,0	83,0	high	(15,17) - (8,5)
ZINC04015296	493,5	750,7	0,0	0,0	9,0	3,3	243,0	107,0	-2,6	0,0	89,0	high	-1,31
ZINC08594547	478,6	807,0	3,0	1,0	8,5	4,3	341,0	154,0	-2,7	0,0	100,0	low	(12,41) - (4,05)
ZINC19924906	453,6	774,9	1,0	0,0	9,0	3,4	391,0	270,0	-3,4	0,0	93,0	high	(6,6) - (0,32) - (-1,91)
ZINC00970517	312,4	521,6	0,0	0,0	1,5	4,9	9906,0	5899,0	0,1	0,0	100,0	low	No ionizable group
ZINC01033612	406,8	692,1	5,0	0,5	5,5	4,4	511,0	590,0	-1972,0	0,0	100,0	low	(12,35) - (9,77) - (2,31)
ZINC08647964	419,4	656,7	4,0	0,0	7,5	2,5	101,0	41,0	-3,5	0,0	77,0	high	(8,92) - (3,82)
ZINC12369546	451,3	709,0	2,0	0,0	4,5	6,0	1918,0	10000,0	-1,4	1,0	100,0	low	No ionizable group
ZINC03120327	393,5	678,2	3,0	0,0	4,0	5,3	1895,0	1410,0	-0,7	1,0	100,0	low	(-0,82)
ZINC19835160	464,6	775,2	2,0	0,0	6,8	4,9	772,0	413,0	-2,8	0,0	100,0	high	(7,11) - (0,89)
ZINC19835187	479,6	746,5	1,0	0,0	7,3	4,8	961,0	883,0	-2,8	0,0	100,0	high	(6,98) - (-0,78)
ZINC19924939	470,6	671,3	2,0	0,0	7,5	3,7	378,0	191,0	-3,4	0,0	95,0	high	(6,35) - (0,27)
ZINC59608258	494,4	737,3	6,0	2,5	5,5	4,7	185,0	698,0	-4,1	0,0	95,0	high	(14,17) - (0,35) - (-1,18)

Table S7. Interactions by H-bond between the compounds and the two protein fragments 3ipk - 3qe5, identified in the pockets established by two meta-servers, using the chimera software.

Compound	Protein	Pockets predictor	Pockets	Energy values (KJ/mol)	N° H bonds	Group of the compound that interacts in the H bond	Amino acid residue that interacts in the H bond	Link distance (Å)
ZINC19835187	3IPK	COACH	1	-10,7	1	Thiazole	Lys 811	3.383
		MET		-10,7	1	Thiazole	Lys 811	3.293
		COACH	2	-9,2	2	4-carbonyl	Asn 820	2.733
						Thiazole	Thr 652	3.407
		MET		-8,8	2	4-carbonyl	THR 837	2.313
						Thiazole	Lys 621	2.895
		COACH	3	-8,5	0			
		MET		-10,3	1	Thiazole	SER 697	3.113
	3QE5	COACH	1	-8,1	2	Thiazole	LYS 1023	2.559

						4-carbonyl	GLY 321	2.308
		MET		-8,7	0			
		COACH	2	-7,5	1	2,3-dihydro-1-benzofuran	Asn 1245	2.201
		MET		-8,4	1	4-carbonyl	Arg 1221	2.162
		COACH	3	-6,9	2	2,3-dihydro-1-benzofuran	Lys 1285	2.272
						Thiazole	Arg 1197	2.294
		MET		-8,8	0			
ZINC19924939	3IPK	COACH	1	-10,4	1	1H-pyrazol	Ser 704	2.407
		MET		-10,4	1	1H-pyrazol	Ser 704	2.394
		COACH	2	-9	1	1H-pyrazol	Asn 590	2.748
		MET		-8,9	1	sulfonyl	Thr 837	1.990
		COACH	3	-7,2	0			
		MET		-8,1	0			
	3QE5	COACH	1	-7,6	0			
		MET		-7,5	0			
		COACH	2	-7,1	0			
		MET		-8	0			
		COACH	3	-6,4	2	1H-pyrazol	Arg 1197	2.465
						1H-pyrazol	Arg 1197	2.008
		MET		-7,9	0			
ZINC19924906	3IPK	COACH	1	-11,1	2	Pirazine	Lys 812	2.279
						Thiazole	Ser 704	2.273
		MET		-11	2	Pirazine	Lys 812	2.245
						Thiazole	Ser 704	2.281
		COACH	2	-7,1	0			
		MET		-8,2	2	Pirazine	Lys 621	2.298
						1-carbonyl	Thr 837	2.090
		COACH	3	-7,1	0			
		MET		-9,2	1	Thiazole	Ser 762	2
	3QE5	COACH	1	-6,9	1	Pirazine	Ile 1157	2.239
		MET		-8,3	0			
		COACH	2	-6,7	0			
		MET		-7,5	1	Pirazine	Arg 1221	2.446
		COACH	3	-6,8	1	Pirazine	Asn 1015	1.971
		MET		-7,5	2	Pirazine	Tyr 1131	2.049
						Thiazole	Asn 1128	2.438

Table S8. Water solubility results for the molecules selected, using a consensus from 3 methods Log S (ESOL), Log S (Ali) and Log S (SILICOS-IT).

	**Log S (ESOL).**	**Solubility mg/ml; mol/l**	**Solubility Class**	**Log S (Ali).**	**Solubility mg/ml; mol/l**	**Solubility Class**	**Log S (SILICOS-IT)**	**Solubility mg/ml; mol/l**	**Solubility Class**	**Final Solubility**	**Final Solubility Class**
ZINC19924906	-5,05	4,02e-03; 8,87e-06	Moderately	-4,99	4,65e-03; 1,02e-05	Moderately	-8,13	3,35e-06; 7,40e-09	Poorly	-6,06	**Moderately soluble**
ZINC19835187	-6,26	2,63e-04; 5,48e-07	Poorly	-6,42	1,84e-04; 3,83e-07	Poorly	-8,75	8,49e-07; 1,77e-09	Poorly	-7,14	**Poorly soluble**
ZINC19924939	-5,61	1,16e-03; 2,46e-06	Moderately	-5,26	2,58e-03; 5,48e-06	Moderately	-7,95	5,28e-06; 1,12e-08	Poorly	-6,27	**Moderately soluble**
